# Parasitic Protists: Diversity of Adaptations to a Parasitic Lifestyle

**DOI:** 10.3390/microorganisms10081560

**Published:** 2022-08-03

**Authors:** Iva Kolářová, Isabelle Florent, Andrea Valigurová

**Affiliations:** 1 Laboratory of Vector Biology, Department of Parasitology, Faculty of Science, Charles University, Albertov 6, 128 44 Prague, Czech Republic; 2 Parasites and Free-Living Protists (UMR7245 CNRS-MNHN, MCAM), Department “Adaptations of Living Organisms”, National Museum of Natural History, CEDEX 05, 75231 Paris, France; 3 Department of Botany and Zoology, Faculty of Science, Masaryk University, Kotlářská 2, 611 37 Brno, Czech Republic

Parasitic protists cause some of the most well-known human and animal diseases such as malaria, toxoplasmosis, amoebic meningitis, sleeping sickness, leishmaniosis, and diarrheal illness of protozoan origin (e.g., amoebiasis, cryptosporidiosis, and giardiasis) [1]. While the nature of the diseases and the transmission modes (e.g., vector-borne, food-/water-borne, by contact or fomites) vary widely among parasitic protists, they generally constitute health management challenges and have a significant impact on the global economy. Given the growing number of emerging and re-emerging diseases caused by parasitic protists, there is an urgent need to implement new strategies in vaccine development and therapeutic interventions. The causative agents of these diseases have evolved a wide range of unique adaptations to parasitism, leading to different strategies of invasion, proliferation, and survival within their hosts’ appropriate niches, as well as transmission modes, which hampers our efforts to control them. These traits allow them to manipulate the host (or its host cells), to modulate or evade the host’s immune responses, and even to use host metabolic processes for their own benefit.

This Special Issue of *Microorganisms* has collected altogether two reviews and eight original research articles (Table 1).

The review by Valigurová and Florent [2] provides up-to-date information about nutrient acquisition and attachment strategies in basal lineages of Apicomplexa. The review focuses on trophozoite stages parasitising mainly invertebrate hosts, such as protococcidia, blastogregarines (e.g., *Siedleckia nematoides*), and *Selenidium* archigregarines from the polychaetes and eugregarines or neogregarines from insect hosts. This review underlines the huge diversity of subcellular organisation and highly specialised adaptations to a parasitic lifestyle in basal lineages of Apicomplexa, while comparing them to medically important species such as *Cryptosporidium*, *Plasmodium*, and *Toxoplasma*. The authors emphasise several questions that need to be answered to help us fully understand the origin and evolution of the parasitism strategies and nutritional adaptations in Apicomplexa.

The other review by Kolářová and Valigurová [3] focuses on the adaptations and evasion strategies of parasitic protists using the example of two medically important parasites, *Cryptosporidium* and *Leishmania*, while discussing their different localisation within host tissue, i.e., epicellular vs. intracellular. The authors describe the dynamic parasite–host interactions that reflect a delicate balance between the host’s defence against the parasite and the rapid development and adaptation of the parasite to newly established conditions. Moreover, they also suggest a definition of epicellular parasitism in protozoa that is lacking in current literature.

This Special Issue further collected eight original research articles describing apicomplexan parasites (*Cryptosporidium*, *Eimeria*, *Plasmodium*), extracellular flagellates (*Giardia*, *Trypanosoma*) and a ciliate (*Tetrahymena*), and *Phytophthora* as an oomycete plant parasite.

Ježková et al. [4] screened 150 nutrias (*Myocastor coypus*) in the Czechia and Slovakia for *Cryptosporidium* species. Nutrias were found to be PCR positive for three *Cryptosporidium* species, but only *Cryptosporidium myocastoris* seemed to be well adapted to this host species. This was supported by the fact that only nutrias positive for *C. myocastoris* shed microscopically detectable oocysts and that these oocysts were infectious for naive nutrias, causing asymptomatic infection.

Zhang et al. [5] characterised *Cryptosporidium parvum* T-cell immunomodulatory protein homolog (CpTIPH) and showed its localisation on the cell surface of *C. parvum* sporozoites. The authors demonstrated that it binds to host cell surface with high affinity and possibly serves in the parasite invasive process as the anti-CpTIPH antibodies were able to partially block the invasion of *C. parvum* sporozoites into host cells.

Ribeiro E Silva et al. [6] focused their study on *Eimeria tenella*, the veterinary important parasite responsible for avian coccidiosis. They analysed the expression pattern of rhoptry kinases that play a key role in host–pathogen interaction in most of the apicomplexan parasites but are poorly studied in this species. The genome-wide analysis revealed higher transcription in extracellular stages, and 7–9 *ropk* were specifically transcribed in merozoites. Further studies are needed to reveal their function.

Oboh and Thomas’s article [7] is the last one dealing with apicomplexan parasites. Their study supports the evidence of two sympatric subspecies, *Plasmodium ovale curtisi* and *Plasmodium ovale wallikeri*, on samples from Gabon, Senegal, Ethiopia, and Kenya, showing further intrasubspecies variability based on the segregating sites and haplotypes of tryptophan-rich protein and small subunit ribosomal RNA genes. The observed variability may affect the translation of *P. ovale* malaria control measures among different countries.

The other three articles deal with extracellular flagellated protists, namely, *Giardia*, *Trypanosoma*, and a ciliate of the genus *Tetrahymena*.

Morales-Luna et al. [8] engineered a fused enzyme of *Giardia intestinalis*, a medically and veterinary important parasite. They fused glucose-6-phosphate dehydrogenase (G6PD) with the 6-phosphogluconolactonase (6PGL), both active in the pentose phosphate pathway. The fused enzyme G6PD::6PGL is active in *Giardia* metabolism, and its structural difference from the human G6PD indicates that the G6PD::6PGL is a potential drug target for novel anti-*Giardia* drugs.

Fialová et al. [9] described a complete life cycle of *Trypanosoma thomasbancrofti*, an avian trypanosome transmitted by Culicine mosquitoes. Experimentally infected *Culex pipens* females established mature infection of *T. thomasbancrofti* in the hindgut. The birds were subsequently successfully infected by the ingestion of infected mosquito guts containing trypanosomes and via the conjunctiva. These results suggest that prediuresis might be the transmission mode of *T. thomasbancrofti*.

Haites et al. [10] focused their study on *Tetrahymena rostrata*, a facultative ciliate parasite of some species of terrestrial molluscs. They tested its potential as a biopesticide of pest slugs. Theronts of *T. rostrata* (the vegetative stages) were able to kill *Deroceras reticulatum* slugs, while staying viable and infective even after prolonged starvation.

Last but not least, Kuhn et al. [11] used the plant parasite, oomycete *Phytophthora nicotianae*, as a model organism to reveal the evolution of the parasite from an autotrophic ancestor involving metabolic adaptation. This genus displays an unusual repertoire of glycolytic enzymes. The authors reveal that in *P. nicotianae* in particular, there is a set of three glucokinase types that are differentially expressed during the parasite life cycle, including plant infection.

In summary, the main goal of this Special Issue was to provide a platform to exchange current knowledge and to demonstrate that parasitism strategies of these organisms are so specific and unique that they need to be addressed individually in search for modern prophylactic strategies as well as diagnostic and therapeutic tools. We believe that this collection put an important piece of knowledge into the complex picture of the wide biological diversity behind the parasitism strategies of protists, illustrating evolutionary trends in successful parasitism, invasion and survival strategies, host–parasite interactions (immune, cellular, metabolic, etc.), virulence mechanisms, and pathogenesis.

## Figures and Tables

**Table 1:** Reviews and original articles collected in this Special Issue highlighting parasites of interest.

Authors	Parasite of Interest	Doi
Valigurová and Florent	Apicomplexa (review)	10.3390/microorganisms9071430
Kolářová and Valigurová	multiple (review)	10.3390/microorganisms9122434
Ježková et al.	*Cryptosporidium myocastoris*	10.3390/microorganisms9040813
Zhang et al.	*Cryptosporidium parvum*	10.3390/microorganisms9051015
Ribeiro E Silva et al.	*Eimeria tenella*	10.3390/microorganisms9081621
Oboh and Thomas	*Plasmodium ovale*	10.3390/microorganisms10061147
Morales-Luna et al.	*Giardia intestinalis*	10.3390/microorganisms9081678
Fialová et al.	*Trypanosoma thomasbancrofti*	10.3390/microorganisms9102101
Haites et al.	*Tetrahymena rostrata*	10.3390/microorganisms9091970
Kuhn et al.	*Phytophthora nicotianae*	10.3390/microorganisms10020281
